# Types of social support and parental acceptance among transfemale youth and their impact on mental health, sexual debut, history of sex work and condomless anal intercourse

**DOI:** 10.7448/IAS.19.3.20781

**Published:** 2016-07-17

**Authors:** Victory Le, Sean Arayasirikul, Yea-Hung Chen, Harry Jin, Erin C Wilson

**Affiliations:** 1 Center for Public Health Research, San Francisco Department of Public Health, San Francisco, CA, USA; 2 Department of Social and Behavioral Sciences, University of California San Francisco, San Francisco, CA, USA; 3 Department of Epidemiology and Biostatistics, University of California San Francisco, San Francisco, CA, USA

**Keywords:** youth, transgender women, HIV, parental acceptance, social support

## Abstract

**Introduction:**

Transfemale youth (TFY) are an underserved and understudied population at risk for numerous poor physical and mental health outcomes, most notably HIV. Research suggests that parental acceptance and social support may serve as protective factors against HIV and other risks for TFY; however, it is unclear whether TFY receive primary social support from parents with or without parental acceptance of their gender identity. This study examines differences in parental acceptance, mental health and the HIV risk factors of history of sex work, age at sexual debut and engagement in condomless anal intercourse between TFY with two types of primary social support – non-parental primary social support (NPPSS) and parental primary social support (PPSS).

**Methods:**

Cross-sectional data collected from 301 TFY from 2012 to 2014 in the San Francisco Bay Area were analyzed to determine differences in parental acceptance, mental health and HIV risk factors between youth with and without PPSS. Univariate statistics and chi-squared tests were conducted to determine if parental acceptance and health outcomes were correlated with type of social support.

**Results:**

Two-hundred fifty-one participants (83.7%) reported having NPPSS, and 49 (16.3%) reported PPSS. Significantly more youth with PPSS reported affirmative responses on parental acceptance items than their NPPSS counterparts. For example, 87.8% of youth with PPSS reported that their parents believed they could have a happy future as a trans adult, compared with 51.6% of youth with NPPSS (*p*<0.001). Fewer participants with PPSS reported symptoms of psychological distress (2.0% vs. 12.5%, *p*=0.057), though this finding was not statistically significant; no significant associations were found between primary social support type and HIV risk factors.

**Conclusions:**

These results suggest that TFY with parental acceptance of their gender identity may be more likely to reach out to their parents as their primary source of social support. Interventions focused on parental acceptance of their child's gender identity may have the most promise for creating parental social support systems in the lives of TFY.

## Introduction

Transfemale youth (TFY) are a population at high risk for HIV in the United States, with prevalence estimates as high as 19% [1]. Studies suggest that parental relationships affect engagement in risk behaviour among young people [2]. However, existing family research with sexual and gender minorities has focused primarily on lesbian, gay and bisexual (LGB) young people, with little data on gender nonconforming and transgender youth. Studies inclusive of TFY often underrepresent this population. For example, the Family Acceptance Project found that lesbian, gay, bisexual and transgender (LGBT) youth who reported low levels of family acceptance were more likely to be depressed, abuse substances, engage in risky sexual behaviour and have suicidal thoughts and attempts [3]. However, in the cohort of 245 participants, only 9% identified as transgender, making generalizations from the study to transgender youth limited. More nuanced examinations of the role of parents and their impact on the lives and health of TFY are needed.

Conflict between parents and their gender nonconforming children stems from the dissonance parents experience between their socialization of the child's gender at birth and the child's emerging gender identity [4]. Despite the presence of conflict within the family, gender nonconforming children need parental support to thrive, but often face parental rejection instead [5]. In a small study of adult transwomen of colour in New York, 55% reported parental rejection, with 40% reporting verbal abuse or physical violence from family members [6]. A study of 295 transgender adults found that, compared with non-trans siblings, transgender siblings reported less perceived social support from family members [7]. The disconnect between parent and child expectations of gender and sexual orientation has been found to lead to decreased social support among LGBT youth, which is associated with increased depressive symptoms, higher rates of victimization and increased suicide attempts, with transyouth being more likely to attempt suicide than their sexual minority peers [8]. However, few studies have addressed the impact of parental support and rejection among TFY specifically. One study of transwomen of colour found that familial rejection led to an increased risk of homelessness [6]. As a result, some TFY turned to sex work to survive [9]. Though the trans sex work community can be an important source of social support, it may also facilitate engagement in high-risk sex work and risk for HIV and other poor health outcomes [10].

Also missing from the research on TFY health are data on parental influence factors associated with parental social support. Research with LGB youth found that parents with unconditional love for their child embraced their child's sexual orientation/identity and as a result felt a closer bond to their child, gaining cognitive flexibility instead of dissonance [11]. Parental acceptance is associated with protective factors against negative health consequences (i.e. drug/alcohol abuse, depression and suicidal attempts/ideation) and promotes positive health outcomes (i.e. healthy self-esteem, general health and higher quality of life) among LGBT youth [3]. Research has also found that parental acceptance results in parents being a primary source of social support for LGB youth [3,12]. For TFY, social support may be as instrumental as parental acceptance in preserving their well-being [6,13]. TFY need support beyond acceptance for things like health insurance, negotiating school and the expectations of teachers and administrators, and accessing transgender health services [14–16]. We hypothesize that parental social support may be closely tied to the ability of parents to accept their TFY's gender identity; hence, parental social support may be as important as acceptance for TFY. Perhaps even more important, TFY may seek social support from parents only when they know their parents accept their gender identity.

This study was conducted to determine whether TFY with parental acceptance of their gender identity reported primary social support from parents. We also assessed whether there were significant differences between those with non-parental primary social support (NPPSS) and those with primary parental social support (PPSS) in psychological distress and the HIV risk factors of a history of sex work, age at sexual debut and condomless anal intercourse. This is a secondary analysis of cross-sectional data from 301 TFY aged 16 to 24 years in the San Francisco Bay Area.

## Methods

### Participants and recruitment

We conducted a secondary analysis using cross-sectional data that were collected with TFY between August 2012 and June 2014. Participants were recruited through peer referral, outreach on social networking sites, in-person community events, and referrals from both community-based organizations and transgender health clinics. Recruitment procedures are described in detail elsewhere [17,18]. Eligibility criteria for the study were self-identification as any gender other than that typically associated with an assigned male sex at birth, 16 to 24 years of age and living in the San Francisco Bay Area. Informed consent was obtained before starting the behavioural survey, which was administered via handheld tablet computers. All study procedures were approved by the Institutional Review Board at the University of California San Francisco. Written consent was obtained from all youth aged 18 years or older. For those who were under 18 years of age, written consent was provided in accordance with a review board waiver of parental consent.

### Measures and data analysis

Social and demographic data measured were age, gender, race and education. Nativity was measured with the question, “Were you born in the U.S.?” Family religiosity was measured by, “How religious is your family?” Self-religiosity was measured by, “How important is religion in your life?” Possible responses for both family and self-religiosity were very religious, somewhat religious, not very religious and not religious at all. Lifetime history of employment was assessed by asking a question with a dichotomous yes/no answer: “Have you ever worked to support yourself?” Income was assessed by asking, “How much money did you make last month (including all sources)?” Childhood living situation was assessed with the question, “What best describes your living situation between birth and age 16?” Response categories were with parents of origin, with other caregiver or family, foster care system, legally adopted family, homeless and on my own, or other. History of housing instability was assessed with two questions. We asked, “Have you ever had unstable housing?” and “Have you ever run away from home and spent one night somewhere other than home when you parents or caregivers did not know where you were?” Responses for these two questions were dichotomous yes/no (0/1).

Current type of social support was measured by asking participants, “From whom do you get the most social support?” Participants who identified parents as their primary source of social support were coded as having PPSS, and participants who identified non-parents (e.g. friend, partners) as their primary source of social support were coded as having NPPSS. Outcomes measured were parental acceptance, psychological distress, history of sex work, age at sexual debut and condomless anal intercourse. Parental acceptance was measured by developing gender minority-specific constructs based on Ryan *et al*.'s work on family acceptance among sexual minority youth [3]. Ten items were measured for this analysis. Parental acceptance items included, for example, “Did any of your parents or caregivers ever talk about your trans identity with you?” and “Did any of your parents or caregivers ever express affection when you first talked about your trans identity?” Responses were dichotomous yes/no (1/0), and a sum score of parental acceptance was totalled. The measure we developed for gender minority-specific parental acceptance had a Cronbach's alpha for parental acceptance of 0.775, indicating internal consistency of the measure with this sample. Psychological distress was measured with the 18-item version of the Brief Symptom Inventory (BSI-18), converting the BSI-18 Global Severity Index (GSI) to *T*-scores and using a validated clinical cut-off of *T*>62 for symptomatic psychological distress in the past seven days [19–22]. The BSI-18 *T*-scores calculated in this study had high internal consistency (Cronbach's alpha=0.91). Lifetime history of sex work was measured by asking, “Have you ever worked as a sex worker?” Responses were dichotomous yes/no (1/0). Age at sexual debut was determined by the question, “How old were you the first time you had consensual sex?” Participants reported their age in years. Condomless anal intercourse was assessed by asking participants about their most recent sexual partners and whether or not they had engaged in condomless anal intercourse with each partner. If participants reported any condomless anal intercourse with any partner, they were coded as having had condomless anal intercourse in the past six months.

Univariate and bivariate analyses were conducted using SAS, version 9.3 (Cary, NC). Chi-squared tests were conducted to assess significant differences between proportions. First, we described the sample's social and demographic data and overall outcomes. Second, we examined outcomes – parental acceptance, psychological distress, history of sex work, sexual debut and condomless anal intercourse – by type of social support: PPSS and NPPSS. We hypothesized that PPSS was associated with positive means scores for parental acceptance. Unadjusted and adjusted models controlling for age group and race were run, with no qualitative differences in *p*-values; therefore, unadjusted models are provided in Table 3.

## Results

### Participant characteristics

Nearly one-quarter of participants (22.6%) were 16 to 19 years old, and 77.4% were 20 to 24 years old (Table 1). The majority of participants (44.2%) identified as female, followed by transgender (32.9%), and genderqueer/fluid/questioning (22.9%). The sample was racially diverse with 36.5% White, 21.9% Latina, 13.0% African American, 6.3% Asian, 7% other race and 15.3% mixed. Most (84.7%) were born in the United States. More than half of the participants (53.0%) reported that religion was not very important. Almost half of youth (45.9%) had at least some college education, and nearly three-fourths (73.4%) had a monthly income of less than US$1000. Many (57.1%) experienced unstable housing in their lifetime. About a quarter (26.7%) reported having a history of sex work, and the most frequent ages in which sexual debut occurred was 13 to 15 and 16 to 17 (28.6 and 28.2%, respectively). About one-third (37.2%) had reported any condomless anal intercourse in the past six months. Participants had a total of 742 sexual partnerships in the past six months, with an average of 3.23 sexual partners per youth. Most sexual partnerships were casual (*n*=532, 71.7%), and the largest proportion identified their sexual partners as heterosexual (*n*=327, 44.1%).

**Table 1:** Demographic characteristics among transfemale youth in the San Francisco Bay Area, 2012 to 2014

Variable	*n* (%)
Age (years)	
16–17	22 (7.3)
18–19	46 (15.3)
20–21	71 (23.6)
22–23	119 (39.5)
24	43 (14.3)
Gender	
Female	133 (44.2)
Transgender female/transwoman/male to female	99 (32.9)
Genderqueer or genderfluid	49 (16.3)
Additional sex or gender	13 (5.3)
Questioning	4 (1.3)
Race	
Hispanic	66 (21.9)
White	110 (36.5)
Black	39 (13.0)
Asian	19 (6.3)
Other	21 (7.0)
Mixed	46 (15.3)
Education	
Less than secondary school	61 (20.3)
Secondary school	102 (33.9)
Some university	105 (34.9)
University grad/grad	33 (11.0)
Nativity	
U.S. born	254 (84.7)
Foreign born	46 (15.3)
Family religiosity	
Very religious	103 (34.4)
Somewhat religious	103 (34.4)
Not very religious/not religious at all	93 (31.1)
Self-religiosity	
Very important	60 (20.0)
Somewhat important	81 (27.0)
Not very important/not important at all	159 (53.0)
Employment	
Ever worked	229 (76.1)
Monthly income (US$)	
0–500	156 (52.3)
501–1000	63 (21.1)
1001–1500	29 (9.7)
1501–2000	21 (7.0)
2000+	29 (8.3)
Childhood living situation	
With parents of origin	246 (81.7)
With other caregiver in family	25 (8.3)
Foster care system	12 (4.0)
Legally adopted family	10 (3.3)
Homeless	7 (2.3)
Other	1 (0.3)
History of housing instability	
Ever unstable housing	172 (57.1)
Ever run away	158 (52.7)
Brief Symptom Inventory	
Psychological distress (*T*>62)	35 (11.6)
History of sex work	
Yes	70 (26.7)
Age (years) at sexual debut	
Never had sex	25 (8.3)
6–12	33 (11.0)
13–15	86 (28.6)
16–17	85 (28.2)
18–19	53 (17.6)
20–24	19 (6.3)
Condomless anal intercourse in past 6 months	112 (37.2)

### Parental acceptance

Parental acceptance in the overall sample was mixed (Table 2). Many participants reported that parents talked about their trans identity with them and that parents supported their gender identity despite feeling uncomfortable (58.8 and 62.1%, respectively). Many participants reported that their parents required other family members to respect them (53.5%), welcomed lesbian, gay, bisexual, transgender and intersex (LGBTI) friends and/or partners to their home (63.1%), and believed their children could have a happy future as a trans adult (56.8%). Conversely, nearly two out of five participants reported that their parents expressed affection when they first talked about their trans identity and that they advocated for them when they were mistreated because of their trans identity (39.9 and 41.5%, respectively). Additionally, fewer participants reported that their parents had not taken them to LGBTI organizations/events or connected them to LGBTI adult role models (18.6 and 15.0%, respectively).

**Table 2:** Parental acceptance among transfemale youth in the San Francisco Bay Area, 2012 to 2014

Parental acceptance	*n* (%)
Item 1: “talked about gender identity with you”	177 (58.8)
Item 2: “expressed affection when you first talked about your gender identity”	120 (39.9)
Item 3: “supported your gender identity despite feeling uncomfortable”	187 (62.1)
Item 4: “advocated for you when mistreated because of your gender identity”	125 (41.5)
Item 5: “required that other family respect you”	161 (53.5)
Item 6: “ever brought you to LGBTI organization or event”	56 (18.6)
Item 7: “connected you to LGBTI adult role model”	45 (15.0)
Item 8: “welcomed LGBTI friends and/or partners to your home”	190 (63.1)
Item 9: “supported your gender expression”	196 (65.1)
Item 10: “believe you could have a happy future as trans adult”	171 (56.8)

### The relationships between type of social support and parental acceptance, mental health, history of sex work and age at sexual debut

Of the 301 participants, 251 (83.4%) reported NPPSS and 49 (16.3%) reported PPSS (Table 3). Among those with NPPSS, 30.9% reported a friend as their primary source of social support, 15% reported a partner and 14.6% reported their chosen family. Other primary sources of social support came from siblings (6.6%), aunts/uncles/grandparents (2.6%), support groups (7.6%), mentors (2%) and other sources (4.3%). Overall, a greater proportion of those with PPSS than of those with NPPSS reported parental acceptance (80% of those with PPSS reported six or more positive responses on parental acceptance items vs. 47.4% of those with NPPSS, *p*=0.003). Table 3 also describes differences in parental acceptance items by type of social support. A greater proportion of those with PPSS than of those with NPPSS reported having parents that expressed affection when they first talked about their gender identity (65.3% vs. 35.5%, *p*<0.001). This relationship was consistent for all but three items. Compared with those who reported NPPSS, participants who reported PPSS were less likely to exhibit psychological distress (*T*>62), but this finding was not statistically significant (2.0% vs. 12.5%, *p*=0.057). No statistically significant differences were observed between type of social support and history of sex work, sexual debut or condomless anal intercourse.

**Table 3:** Differences in parental acceptance, mental health, history of sex work and age at sexual debut by type of primary social support among transfemale youth in San Francisco, 2012 to 2014

	Social support type			
			
Parental acceptance	Non-parent (*n*=251)	Parent (*n*=49)	Chi-squared	*p*
Item 1	144 (58.1)	33 (67.3)	1.1	0.293
Item 2	88 (35.5)	32 (65.3)	13.9	<0.001
Item 3	150 (60.5)	37 (75.5)	3.3	0.067
Item 4	88 (35.5)	36 (73.5)	22.7	<0.001
Item 5	123 (49.6)	38 (77.6)	11.8	0.001
Item 6	41 (16.5)	15 (30.6)	4.4	0.035
Item 7	33 (13.3)	12 (24.5)	3.2	0.076
Item 8	146 (58.9)	44 (89.8)	15.7	<0.001
Item 9	150 (60.5)	46 (93.9)	18.9	<0.001
Item 10	128 (51.6)	43 (87.8)	20.4	<0.001
Total positive (i.e. yes) responses for parental acceptance items				
0–1	47 (27.2)	0 (0.0)	16.1	0.003
2–3	21 (12.1)	3 (8.6)		
4–5	23 (13.3)	4 (11.4)		
6–8	65 (37.6)	21 (60.0)		
9–10	17 (9.8)	7 (20.0)		
HIV risk				
History of sex work	59 (27.4)	10 (22.7)	0.2	0.647
Age (years) at sexual debut				
Never had sex	18 (7.3)	4 (8.2)	4.6	0.466
6–12	28 (11.3)	16 (32.7)		
13–15	70 (28.2)	16 (32.7)		
16–17	68 (27.4)	5 (10.2)		
18–19	48 (19.4)	2 (4.1)		
20–24	16 (6.5)	6 (12.2)		
Condomless anal intercourse	92 (37.1)	18 (36.7)	0.0	1.000
Mental health				
Brief symptom inventory (*T*>62)	31 (12.5)	1 (2.0)	3.6	0.057

## Discussion

Parental acceptance may be the key to increasing parental social support systems for TFY. In our data, we found that TFY who reported having PPSS experienced greater parental acceptance than those who reported NPPSS. Many LGBT youth may not disclose their identity to their parents out of fear [23] and anticipated lack of acceptance. Such fears are relevant for TFY, as about half of youth in our study whose parents did know about their gender identity reported their parents were not accepting of their trans identity.

Interventions to increase parental acceptance of TFY may positively affect TFY's access to parental social support, which could have protective effects on preventing risks for HIV and other poor health outcomes. A study of parental social support for TFY was associated with regular condom use during sex, while youth without parental social support reported less consistent condom use [1]. In research with other youth populations, emotional support from parents was associated with more consistent condom use [24,25] and sexual health [26]. Research suggests that parental social support is important to the gender transition of trans youth [27]. PPSS may also be protective of HIV risk for TFY; however, like in research with LGBT youth [3], we did not see any associations between sexual risk and types of primary social support. More research that looks for such associations over time may be better at detecting the importance of PPSS on risk behaviour in this population.

Our data also suggest that PPSS may affect mental health. We found that psychological distress was observed in greater proportions among those with NPPSS than among those with PPSS. TFY without PPSS may be more likely to experience psychological distress resulting in increases in mental health disorders over the life course. Interventions to increase parental acceptance and PPSS may serve to reduce risk for mental health disorders.

This study was not without limitations. It was conducted in the San Francisco Bay Area and did not use a random sampling approach; therefore, results cannot be generalized to the population of TFY. It is also possible that youth in our sample had parental support but did not get categorized as such because they did not list parents as their *primary* source of support. Measuring parents as a primary source of support is a limitation in the way our data were collected, but we believe this analysis approach fits our investigation given the importance of parents as sources of sexual health information and healthy development. The directionality and associations cannot be further examined because the data are cross-sectional. Further, we did not use mediation hypotheses that may address mechanisms more specifically related to PPSS and health outcomes in this population. Lastly, the cross-sectional nature of the data limits our ability to determine if support can precede parental acceptance. However, our working emic theory is that parents are limited in their ability to provide their trans child support if they are not able to first accept the child's gender identity. This is one of very few studies that delved into understanding the role of parents in HIV risk for TFY and as such provides an important building block to future research.

Understanding the importance of parental acceptance in concordance with PPSS needs to be researched further in order to create interventions for parents to help connect and guide their transgender children. Additionally, services that focus on family support may provide important protective factors for TFY. A national report on homelessness among LGBT youth highlights promising interventions, including LGBT-affirming family counselling for runaway youth and their families [28]. Education for parents on ways to be accepting and supportive of their trans children is urgently needed so parents can develop skills for connecting and supporting their children through important gender milestones.

## Conclusions

The results from this study provide evidence of a link between primary social support from parents and parental acceptance. These data confirm our hypothesis that parental acceptance and parents as the primary source of social support are important factors in TFY's development. Future studies examining the relationship between changes in PPSS and changes in parental acceptance and health over time are needed to understand mechanisms that affect the HIV risk of TFY. Interventions focused on parental acceptance of their children's gender identity may have the most promise for creating PPSS systems for TFY.
